# New record of *Hymenophyllumcaudatum* Bosch (Polypodiopsida, Hymenophyllaceae) extends the mainland distribution in the coastal Mediterranean Forest of South America.

**DOI:** 10.3897/BDJ.10.e84169

**Published:** 2022-08-03

**Authors:** Jimmy Pincheira-Ulbrich, Ulises Zambrano, Jonathan Urrutia-Estrada

**Affiliations:** 1 Universidad Católica de Temuco, Departamento de Ciencias Ambientales, Laboratorio de Planificación Territorial, Rudecindo Ortega 02950, Temuco, Chile Universidad Católica de Temuco, Departamento de Ciencias Ambientales, Laboratorio de Planificación Territorial Rudecindo Ortega 02950, Temuco Chile; 2 Universidad Católica de Temuco, Facultad de Recursos Naturales, Geografía, Temuco, Chile Universidad Católica de Temuco, Facultad de Recursos Naturales, Geografía Temuco Chile; 3 Laboratorio de Invasiones Biológicas, Facultad de Ciencias Forestales, Universidad de Concepción, Concepción, Chile Laboratorio de Invasiones Biológicas, Facultad de Ciencias Forestales, Universidad de Concepción Concepción Chile

**Keywords:** stream flora, filmy fern, species distribution, species inventory

## Abstract

During a botanical exploration in the Los Ruiles National Reserve (Chile), a population of *Hymenophyllumcaudatum* Bosch was identified. Fronds were found at the base of a rock, under a hygrophilous vegetation cover, in a ravine (35°49'56.49"S -72°30'42.44"W). The finding in this wilderness area extends the distribution by 120 km northwards on the mainland, which until now was limited to the coastal area of the city of Concepción (36°47'07.86"). This contribution presents an observed specimen, the site of the find and the accompanying species.

## Introduction

*Hymenophyllumcaudatum* Bosch is one of 24 species of filmy ferns described for the temperate forests of South America. In insular Chile, the species is found in the Juan Fernández Archipelago (e.g. Furniel 2018) and on Mocha Island (e.g. Reiche 1903), more than 600 km and 32 km from the mainland, respectively. The known range of this species on the continent places the northern distribution limit on the Alejandro Selkirk (33°44'39.17''S) and Robinson Crusoe (33°38'34.33''S) Islands in the Archipelago above (Valparaiso Region), while on the mainland, the northernmost record is located on the coastal strip of Concepción city (Parque Hualpén, Biobío Region, 36°47'07.86''S). The southern limit is found at Puerto Edén, on Wellington Island, Magallanes Region (49°09'S -74°26'20"W) (pers. comm. Alicia Marticorena, curator of the CONC Herbarium, see also Diem and Lichtenstein 1959, Rodríguez 1995, Rodríguez et al. 2009, Larsen et al. 2013). In Argentina, the species occurs in the Province of Chubut, in Lago Puelo National Park (e.g. Cassá De Pazos et al. 2010) and Los Alerces National Park (see Larsen et al. 2013). The wide range of the species determines its occurrence in a Mediterranean-temperate transition zone to the north and anti-boreal climate to the south (Luebert and Pliscoff 2006).

A similar species with comparable morphology is found in the Atlantic Forest of Brazil, around 2000 km distant. Although precedents were arguing for morphological differences between the species found in Chile and Brazil (e.g. Diem and Lichtenstein 1959, Rodríguez 1995, Ebihara et al. 2006), evidence was insufficient to classify these populations as two separate species. However, based on genetic and morphological traits, Larsen et al. (2020) propose the name *H.caudatum* for the species found in the temperate forests of Chile and Argentina, while the species growing in the tropical and subtropical forests of Brazil would retain the original name given to the species: *Hymenophyllumcaudiculatum*.

*H.caudatum* inhabits very humid and shady mature forests. In general, it has an epiphytic habit on trunks. However, it is also possible to observe it on the ground or on rocks (Larsen et al. 2013, Furniel 2018), even on decaying logs (e.g. Pincheira-Ulbrich et al. 2021).

## The new record

**Figure 1.**
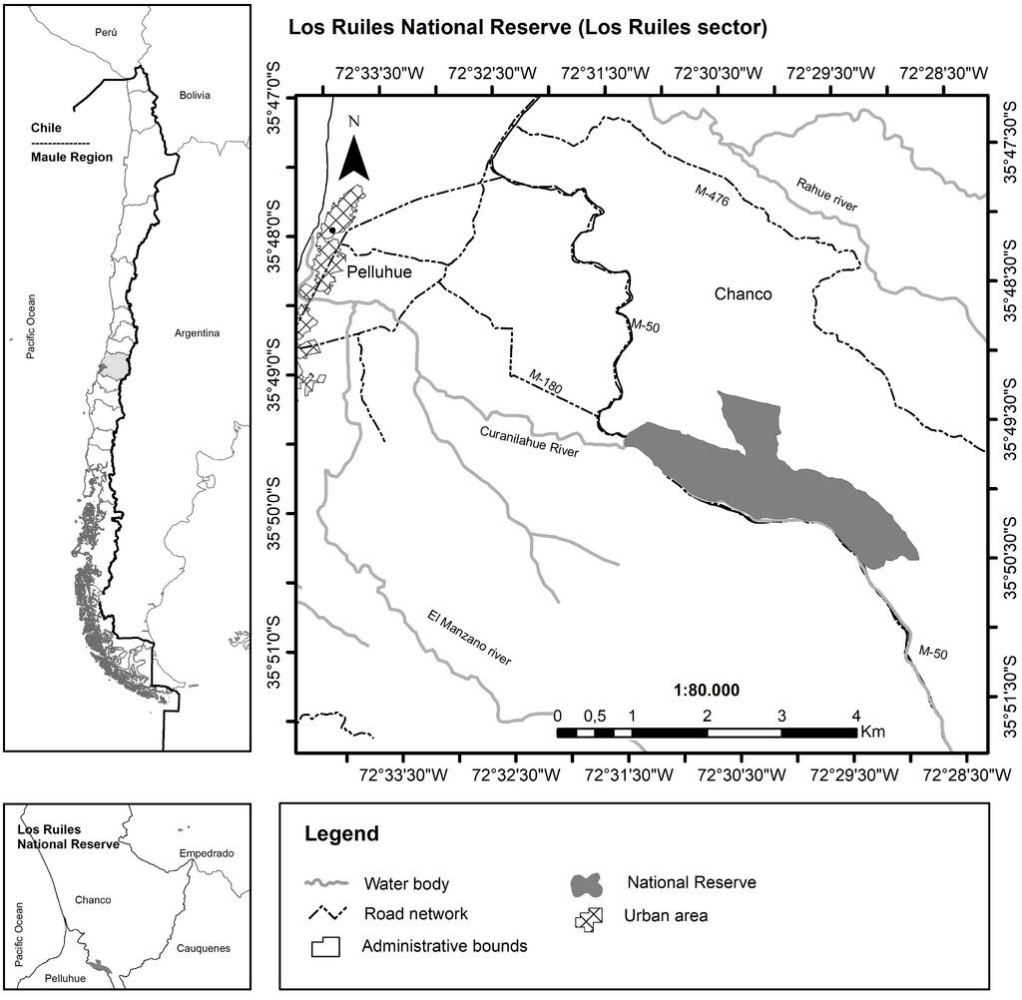
Location map of Los Ruiles National Reserve.

In a botanical exploration conducted on 14 September 2021 in the Los Ruiles sector of Los Ruiles National Reserve (Maule Region, Chile; Fig. 1), ten fronds of *H.caudatum* were observed in a small ravine (35°49'55.58"S; 72°30'42.55"W). The species was found on the southeast face and at the base of a partially moss-covered rock at 228 m above sea level (Fig. 2, Fig. 3). The finding in this wilderness area extends the distribution by 120 km northwards on the mainland, which until now was limited to the coastal area of Concepción city (36°47'07.86") (e.g. Rodríguez 1995, Rodríguez et al. 2009). The sample was deposited in Universidad de Concepción's Herbarium under code CONC 192213.

The site is located about 240 m on a straight line from the main access to the Reserve, surrounded by a forest of *Nothofagusglauca* Krasser. To reach the site, a footpath, not open to the public, is followed before turning off along a ravine (Fig. 2).

On the same rock, we counted 57 fronds of *Hymenophyllumtunbrigense* (L.) Sm., two rosettes of *Fasciculariabicolor* (Ruiz & Pav.) Mez and one individual of *Lapageriarosea* Ruiz & Pav, with a climbing habit, fallen on the rock from a culm of *Chusqueaculeou* E. Desv. In a radius of three metres from the centre of the rock, *Persealingue* (Ruiz & Pav.) Nees (DBH = 10 cm) and *Aextoxiconpunctatum* Ruiz & Pav. (DBH = 32 cm) were present in the tree layer and *Cryptocaryaalba* (Molina) Looser was present on the forest floor at the regeneration stage. In the shrub stratum, *Azarapetiolaris* (D.Don) I.M.Johnst., *Jovellanapunctata* Ruiz & Pav., *Rhamnusdiffusus* Clos., *Baccharisracemosa* DC. and *Ugnicandollei* (Barnéoud) O. Berg were observed. On the herbaceous layer, *Adiantumchilense* Kaulf., *Chusqueacoleu*, *Dioscoreabridgesii* Griseb. ex Kunth, *Nassella* spp. and *Greigiasphacelata* (Ruiz & Pav.) Regel were present. The climber *Lardizabalabiternata* Ruiz & Pav. was also recorded at a diameter of more than 1 cm on an individual of *A.punctatum* (Table 1).

### Importance for conservation

This finding highlights the importance of protecting wetlands to maintain biodiversity (Möller and Muñóz-Pedreros 2014), especially the remaining Mediterranean Forest ravines in the landscape, particularly in the context of climate change (Peñuelas et al. 2017). For example, Troncoso and San Martín (1988) found new populations of vascular plants in small *Drimyswinteri* J.R.Forst. & G.Forst forests located in a series of ravines in a nearby geographical area, which implied an extension of the northern limit of the range for several of these species. Similarly, Stoll and Hahn (2004) extended the northern limit of three species of the Hymenophyllaceae family, recorded in two ravines of the coastal mountain range in the same area. These species were: *Hymenophyllumcruentum* C.Presl, found growing on rocks, *Hymenophyllumdarwinii* Hook.f. ex Bosch, growing epiphytically and *Trichomanesexsectum* Kunze found on the rock wall of a cave. Therefore, it seems necessary to encourage the development of inventories and basic research in streams or forest remnants, as well as to promote the training of advanced human capital in botany, taxonomy and genetics, which has been scarcely encouraged by the Chilean State.

## Figures and Tables

**Figure 2a.**
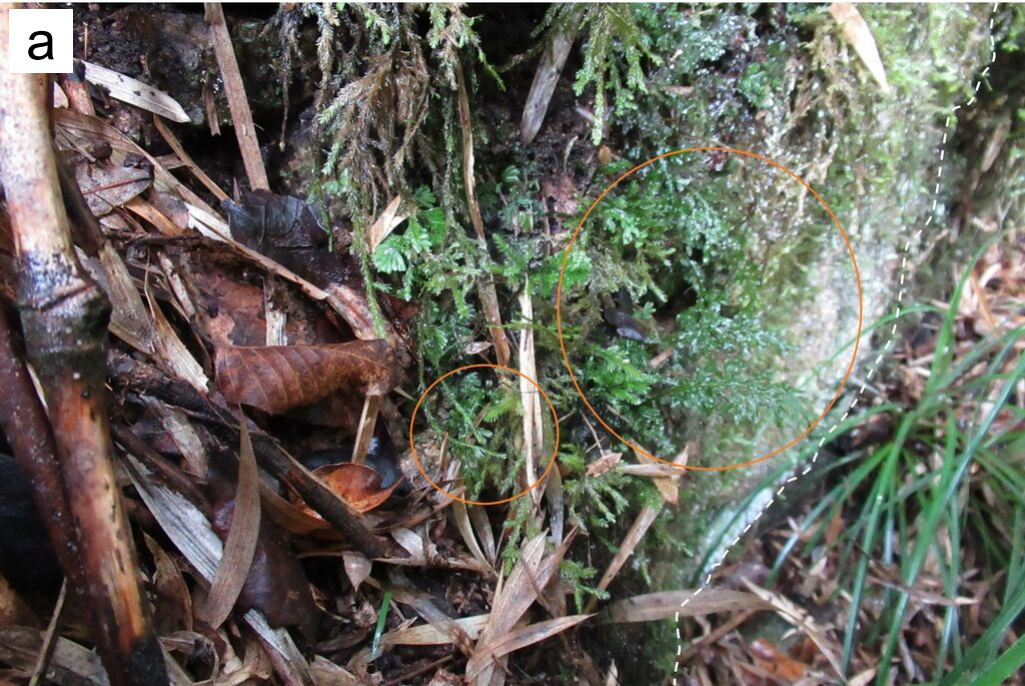
Accompanying species: *Hymenophyllumtunbrigense*, *Chusqueaculeou* and also a single leaf of *Nothofagusglauca*.

**Figure 2b.**
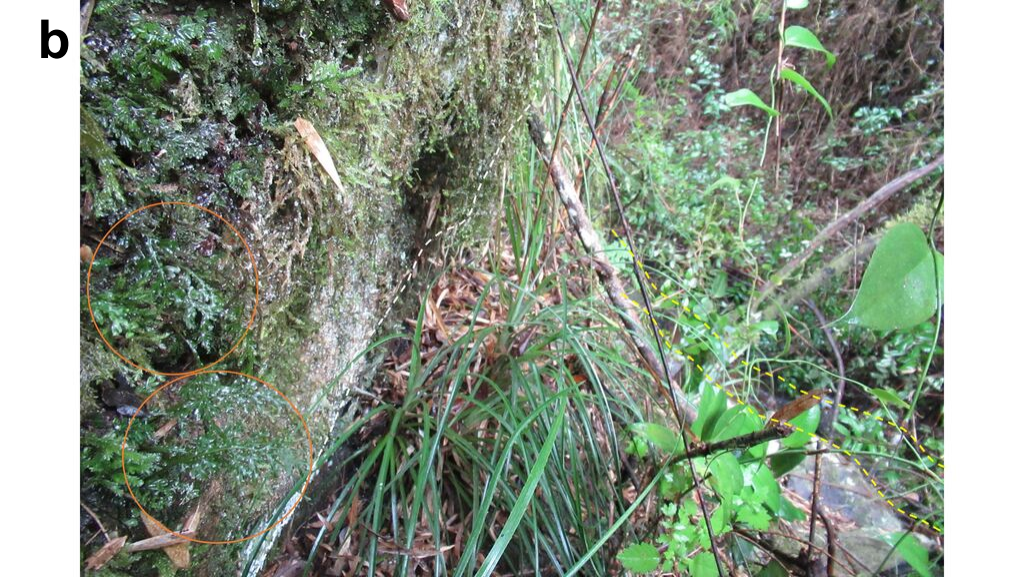
Accompanying species: *Fasciculariabicolor*, *Jovellanapunctata* and *Lapageriarosea*.

**Figure 3a.**
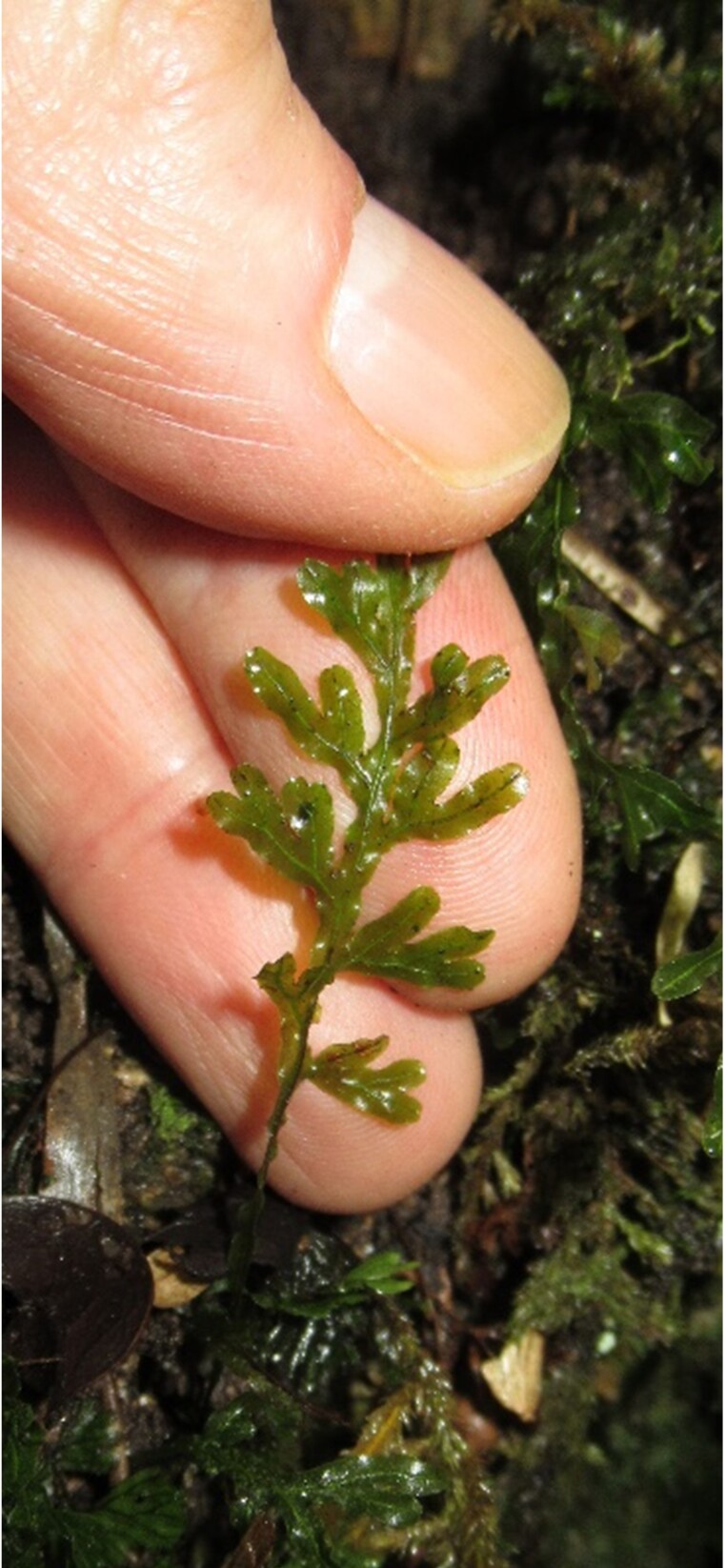
Frond

**Figure 3b.**
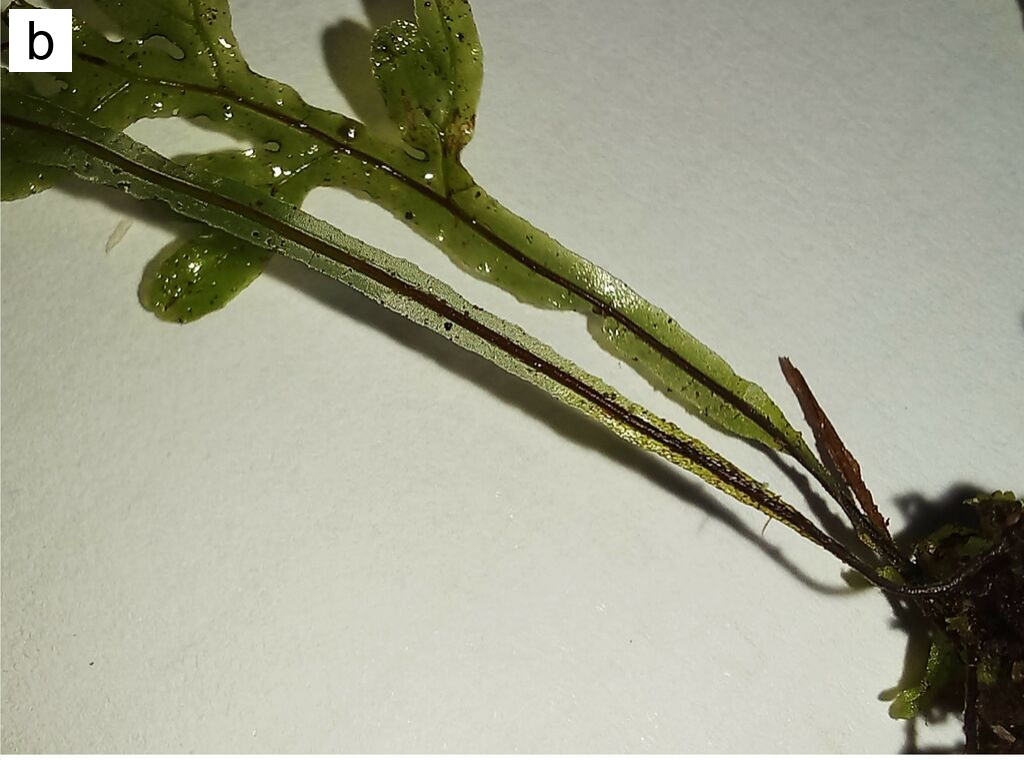
Petiole and rachis

**Figure 3c.**
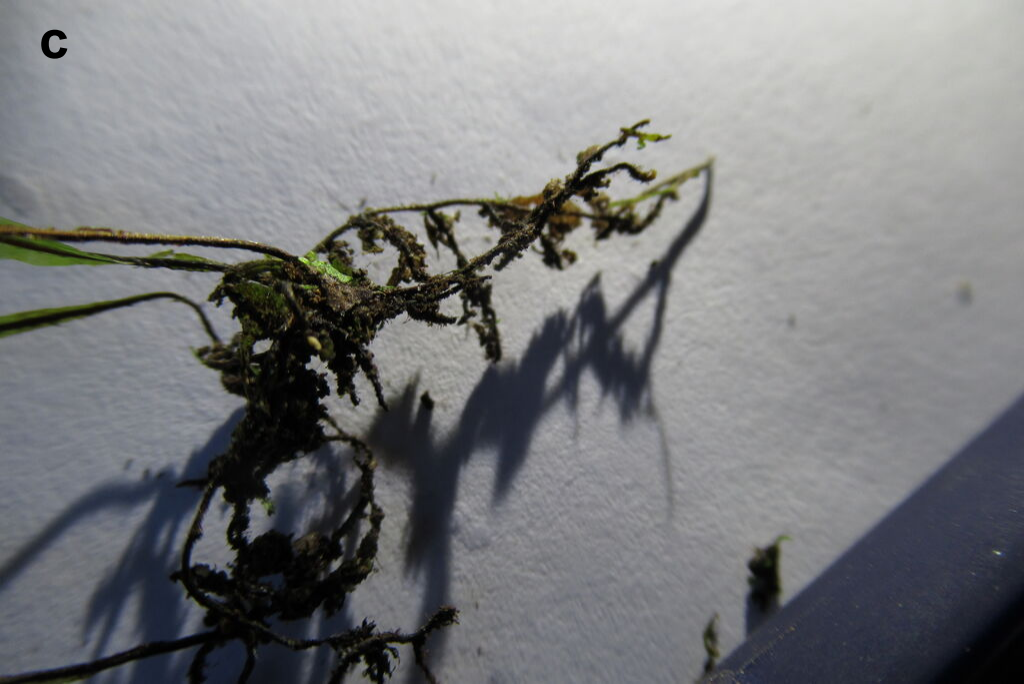
Rhizome

**Figure 3d.**
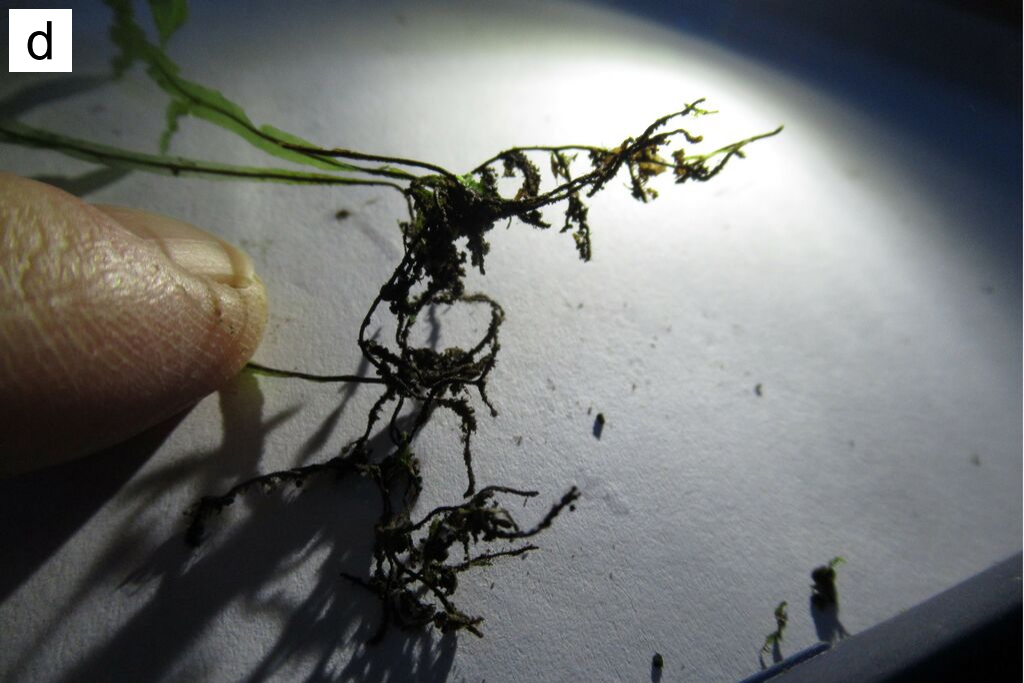
Petiole and rhizome (human finger scale)

**Figure 3e.**
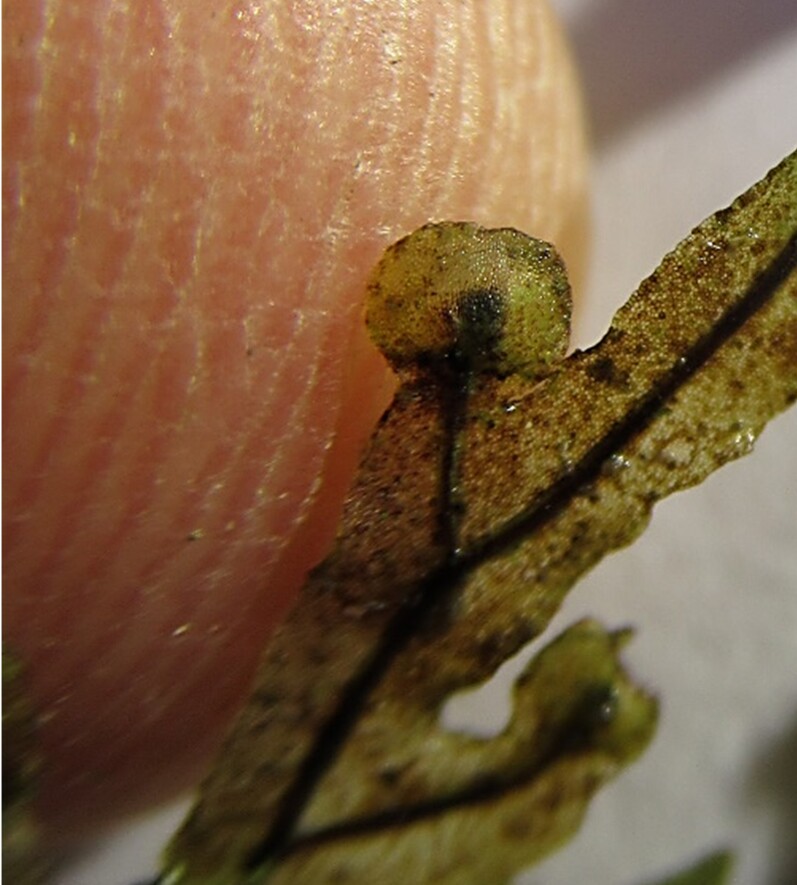
Sorus (human finger scale)

**Figure 3f.**
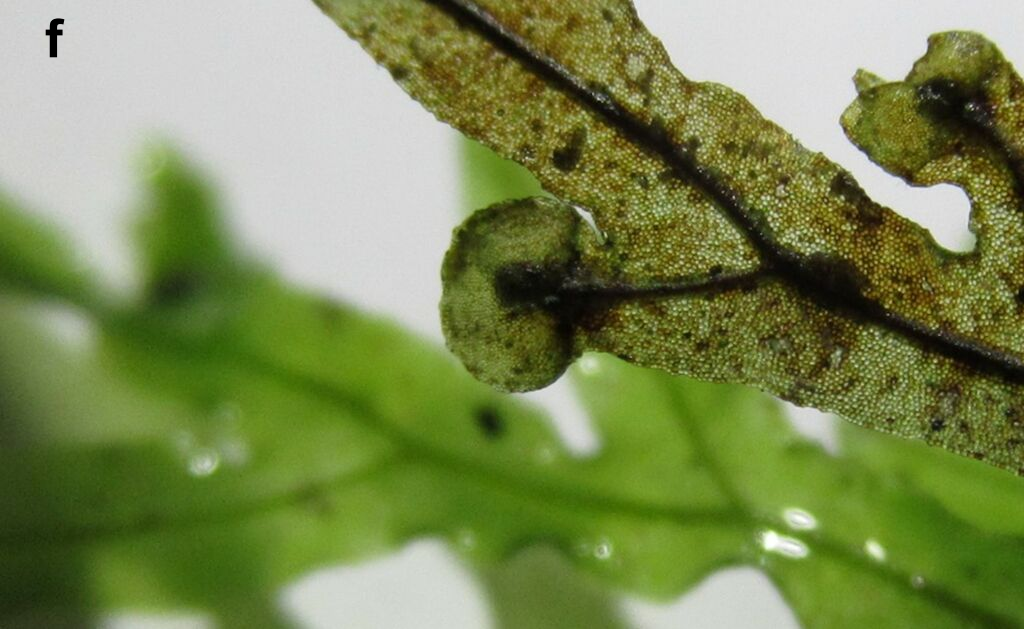
Sorus

**Table 1.** Companion species found in the surroundings of *H.caudatum*. Species classification according to family, growth form and habit criteria are based on Rodriguez et al. (2018) and IPNI (2022).

Specie	Family	Gowth form	Habit
*Adiantumchilense* Kaulf.	Pteridaceae	Terricolous	Herb
*Aextoxiconpunctatum* Ruiz & Pav.	Aextoxicaceae	Terricolous	Tree
*Azarapetiolaris* (D.Don) I.M.Johnst.	Salicaceae	Terricolous	Shrub
*Baccharisracemosa* DC.	Asteraceae	Terricolous	Shrub
*Chusqueaculeou* E. Desv.	Poaceae	Terricolous	Herb
*Cryptocaryaalba* (Molina) Looser	Lauraceae	Terricolous	Tree
*Dioscoreabridgesii* Griseb. ex Kunth	Dioscoreaceae	Terricolous / Vine	Herb
*Fasciculariabicolor* (Ruiz & Pav.) Mez	Bromeliaceae	Lithophyte / Epiphyte	Herb
*Greigiasphacelata* (Ruiz & Pav.) Regel	Bromeliaceae	Terricolous	Herb
*Hymenophyllumtunbrigense* (L.) Sm.	Hymenophyllaceae	Lithophyte / Epiphyte	Herb
*Jovellanapunctata* Ruiz & Pav	Calceolariaceae	Terricolous	Shrub
*Lapageriarosea* Ruiz & Pav	Philesiaceae	Terricolous / Vine	Shrub
*Lardizabalabiternata* Ruiz & Pav.	Lardizabalaceae	Terricolous / Liana	Shrub
*Nassella* spp.	Poaceae	Terricolous	Herb
*Persealingue* (Ruiz & Pav.) Nees	Lauraceae	Terricolous	Tree
*Rhamnusdiffusus* Clos	Rhamnaceae	Terricolous	Shrub
*Ugnicandollei* (Barnéoud) O. Berg	Myrtaceae	Terricolous	Shrub

## References

[B7787100] Cassá De Pazos L., Vidoz F., Giudice G., Ramos J., Luna M., De La Sota E. (2010). Diversidad de helechos y licofitas del Parque Nacional Lago Puelo (Chubut-Argentina). Boletín de la Sociedad Argentina de Botánica.

[B7886138] Diem J., Lichtenstein J. (1959). Las Himenofiláceas del área argentino-chilena del sud. Darwiniana.

[B7887917] Ebihara A., Dubuisson J., Iwatsuki K., Hennequin S., Ito M. (2006). A Taxonomic Revision of Hymenophyllaceae. Blumea - Biodiversity, Evolution and Biogeography of Plants.

[B7787169] Furniel P. (2018). Flora Vascular Silvestre del Archipiélago Juan Fernández.

[B7889229] IPNI International plant names index. Published on the Internet. The RoyalBotanic Gardens, Kew, Harvard University Herbaria & Libraries and Australian National Botanic Gardens.. http://www.ipni.org.

[B7787091] Larsen Cristian, Ponce M. Mónica, Scataglini M. Amalia (2013). Revisión de las especies de *Hymenophyllum* (Hymenophyllaceae) del sur de Argentina y Chile. Gayana. Botánica.

[B7886120] Larsen C., Gonzatti F., Acosta J., Pince M. (2020). Morphological and Molecular Evidence to Segregate a Disjunct Species of Hymenophyllum (Hymenophyllaceae) from Southern South America. Systematic Botany.

[B7787187] Luebert F., Pliscoff P. (2006). Sinopsis bioclimática y vegetacional de Chile.

[B7886448] Möller P., Muñóz-Pedreros A. (2014). Legal protection assessment of different inland wetlands in Chile. Revista Chilena de Historia Natural.

[B7889108] Peñuelas J., Sardans J., Filella I., Estiarte M., Llusià J., Ogaya R., Carnicer J., Bartrons M., Rivas-Ubach A., Grau O., Peguero G., Margalef O., Pla-Rabés S., Stefanescu C., Asensio D., Preece C., Liu L., Verger A., Barbeta A., Achotegui-Castells A., Gargallo-Garriga A., Sperlich D., Farré-Armengol G., Fernández-Martínez M., Liu D., Zhang C., Urbina I., Camino-Serrano M., Vives-Ingla M., Stocker B. D., Balzarolo M., Guerrieri R., Peaucelle M., Marañón-Jiménez S., Bórnez-Mejías K., Mu Z., Descals A., Castellanos A., Terradas J. (2017). Impacts of Global Change on Mediterranean Forests and Their Services. J. Impacts of Global Change on Mediterranean Forests and Their Services. Forests.

[B7787111] Pincheira-Ulbrich Jimmy, Vallejos Bárbara, Huincaguelo Jorge, Zambrano Ulises, Peña-Cortés Fernando (2021). A 30-year update of the climbers and vascular epiphytes inventory of the Cerro Ñielol Natural Monument (La Araucanía, Chile): a database. Biodiversity Data Journal.

[B7787121] Reiche C. (1903). La isla de la Mocha: estudios monográficos bajo la cooperación de F. Germain, M. Machado, F. Philippi y I. Vergara. Anales del Museo Nacional de Chile.

[B7889179] Rodriguez R., Marticorena C., Alarc&oacute n D., Baeza C., Cavieres L., Finot V., Fuentes N., Kiessling A., Mihoc M., Pauchard A., Ruiz E., Sanchez P., Marticorena A. (2018). Catálogo de las plantas vasculares de Chile. Gayana. Botánica.

[B7787147] Rodríguez R., Rodríguez R. (1995). Flora de Chile: Vol. 1. Pteridophyta - Gymnospermae.

[B7787130] Rodríguez R., Alarcón D., Espejo J. (2009). Helechos nativos del centro y sur de Chile. Guía de campo.

[B7886482] Stoll A., Hahn S. (2004). Nuevos registros extienden distribuciones de tres especies de Hymenophyllaceae (Pteridophyta) a la Región del Maule, Chile. Gayana Botánica.

[B7886473] Troncoso A., San Martín J. (1988). Ampliación de área para diversas especies de plantas vasculares en la Cordillera de la Costa de la Región del Maule. Boletín del Museo Nacional de Historia Natural.

